# Immunometabolic cross-talk in the inflamed heart

**DOI:** 10.15698/cst2019.08.194

**Published:** 2019-06-07

**Authors:** Federica M. Marelli-Berg, Dunja Aksentijevic

**Affiliations:** 1 William Harvey Research Institute, Queen Mary University of London, Charterhouse Square, London EC1M 6BQ, United Kingdom.; 2 School of Biological and Chemical Sciences, Queen Mary University of London, G.E. Fogg Building, Mile End Road, London E1 4NS, United Kingdom.; 3 Centre for Inflammation and Therapeutic Innovation, Queen Mary University of London, Charterhouse Square, London EC1M 6BQ, United Kingdom.

**Keywords:** heart, inflammation, metabolism, innate immune response, adaptive immune responses

## Abstract

Inflammatory processes underlie many diseases associated with injury of the heart muscle, including conditions without an obvious inflammatory pathogenic component such as hypertensive and diabetic cardiomyopathy. Persistence of cardiac inflammation can cause irreversible structural and functional deficits. Some are induced by direct damage of the heart muscle by cellular and soluble mediators but also by metabolic adaptations sustained by the inflammatory microenvironment. It is well established that both cardiomyocytes and immune cells undergo metabolic reprogramming in the site of inflammation, which allow them to deal with decreased availability of nutrients and oxygen. However, like in cancer, competition for nutrients and increased production of signalling metabolites such as lactate initiate a metabolic cross-talk between immune cells and cardiomyocytes which, we propose, might tip the balance between resolution of the inflammation versus adverse cardiac remodeling. Here we review our current understanding of the metabolic reprogramming of both heart tissue and immune cells during inflammation, and we discuss potential key mechanisms by which these metabolic responses intersect and influence each other and ultimately define the prognosis of the inflammatory process in the heart.

## INTRODUCTION

Metabolic reprogramming of immune cells during tissue inflammation has been intensely investigated as reviewed elsewhere [1–5]. Notably, three key concepts have emerged from these studies. First, the effector functions of immune cells depend on metabolic reprogramming in response to activation. For example, resting immune cells utilize energetically efficient processes such as the Kreb's cycle linked to the generation of adenosine triphosphate (ATP) via oxidative phosphorylation (OXPHOS) [2] and shift to aerobic glycolysis in response to inflammatory stimuli [6]. Second, the metabolic status of the whole body has been shown to affect functional and metabolic responses of both immune cells and organs. For example, the microbiome and metabolites produced by commensal bacteria are known to affect immune cells as well as organ homeostasis [7] and systemic metabolic diseases such as diabetes and obesity can adversely impact immunity and the function of a variety of organs [7]. Finally, the metabolic adaptation of immune cells is strongly affected by the microenvironment of the inflammatory site, in terms of nutrient availability, oxygen tension and the production of signalling metabolites by the diseased tissue itself. Cancer is paradigmatic of this effect, whereby aberrant metabolic responses by immune cells in response to the tumour microenvironment result in the induction of immune paralysis and cancer survival and expansion [8]. Similar observations have been made in the context of chronic inflammation [9].

While much is known about the metabolic configuration of immune cells in homeostasis and inflammation as well as the effects of systemic metabolism on immune cell function, the metabolic cross-talk between immune cells and organ parenchymal cells has been a challenging issue to address. This is mainly due to the complexity of defining the relative contribution of different cellular components to the metabolic microenvironment on effector immunity and vice versa. Unravelling the influence of the tissue microenvironment on the cross-talk between immune and parenchymal cells during inflammation leading to tissue damage and organ failure is vital to identify prognostic biomarkers and novel targets for therapeutic intervention. A vast amount of reported observations has associated metabolic syndrome – a heart-extrinsic factor - with heart functional impairment as well as systemic sub-clinical chronic inflammation, pointing to metabolic dysfunction as a key determinant of inflammatory heart disease.

In this review, we summarize key concepts in metabolic adaptation in the heart and immune cells in steady-state and inflammation. We highlight cardiac inflammation as a condition in which well-defined systemic and cellular components and metabolic pathways can provide a new paradigm to understand the reciprocal regulation of immune cell and tissue metabolism in inflammatory diseases.

## CAVEATS AND DEFINITIONS: WHAT IS INFLAMMATORY DISEASE OF THE HEART MUSCLE?

An enormous amount of data is available on the link between metabolic syndrome, inflammation and atherosclerosis, including coronary artery disease (CAD). A number of clinical trials has examined the effects of anti-inflammatory therapy on these conditions, with mixed outcomes as their interpretation is hugely varying [10–12]. This topic has been extensively reviewed elsewhere [10–12]. While CAD can indirectly affect cardiac function and metabolism, this review focuses on the direct effects of systemic inflammation on heart muscle inflammation (myocarditis), function and metabolism. Atherosclerosis is an inflammatory disease of the vasculature which impacts both cardiac function and energetics, which is however distinct from inflammation of the heart muscle itself. Myocardium inflammation is commonly associated with an acute viral infection of the heart which normally resolves but can sometimes persist as an autoimmune condition, leading to heart muscle dysfunction and ultimately, heart failure (HF). Myocarditis is notoriously difficult to diagnose, and its viral aetiology appears to account for only a fraction of clinically diagnosed heart inflammation. A key concept recently pioneered by Heymans and colleagues is that besides direct inflammatory responses in the heart, such as those detected in viral myocarditis, genetic predisposition in association with environmental factors can initiate and sustain cardiac inflammation leading to heart failure [13]. About 40% of acute HF is diagnosed as idiopathic/inflammatory dilated cardiomyopathy (iDMC). This alternative definition is not necessarily based on histological evidence, as endomyocardial biopsy has limited sensitivity [14]. Overall, persisting cardiac inflammation appears to be a feature of a large proportion of heart conditions beyond viral acute myocarditis. Typical examples include ischemic cardiomyopathy and HF with preserved ejection fraction (HFpEF) and, more recently fulminant myocarditis as a serious adverse reaction of cancer therapies that enhance T cell responses [15]. Although treatment has dramatically improved survival post-myocardial infarction (MI), more than 20% of patients subsequently develop cardiomyopathy and HF. Ischemia-induced cardiomyocyte death activates an inflammatory response that serves to clear the injured myocardium from dead cells, and stimulates repair.

However, cardiomyocyte necrosis is also a powerful initiator of autoimmune inflammation of the heart leading to HF (reviewed in [16]). Interleukin-1(IL-1) α and RNA released by necrotic cardiomyocytes are key danger signals that trigger the inflammatory response following MI (reviewed in [17]). IL-1 induces a proinflammatory phenotype in leukocytes and fibroblasts, and delays myofibroblast transdifferentiation. Anti-inflammatory regulatory T cells (Tregs) exert negative regulation of the inflammatory response post-MI by modulating macrophage and fibroblast phenotype. Cardiac macrophages exhibit significant heterogeneity and phenotypic plasticity and may orchestrate the reparative response following infarction [17]. The persistence of inflammation can cause immune-mediated tissue damage [16] and progressive structural changes such as left ventricular (LV) remodeling and functional impairment [18–20]. Collectively, these studies reveal a complex cross-talk between cells of the immune system and the myocardial parenchyma, which likely determines the fate of the inflammatory process. Similarly, systemic inflammation, and in particular that induced by metabolic syndrome, can drive disease of the myocardium. For example, there is rising consensus that HFpEF is primarily driven by inflammation induced by ageing and related comorbidities, including obesity and diabetes [21, 22]. In line, therapeutic interventions with anti-inflammatory properties have been also shown to protect from experimental HFpEF [23]. A link between systemic inflammation and cardiac has also been reported by studies correlating systemic inflammation markers (ie. cytokines) with well-known cardiac arrhythmias including atrial fibrillation and ventricular tachycardia [24]. Despite this evidence, diagnostic and prognostic biomarkers of cardiac inflammation and, importantly, mechanistic studies are missing. We propose that disturbances in the metabolic configuration of the heart muscle, with its lifetime of varying workload and energy demand, are induced by direct and indirect cross-talk with inflammatory immune cells via both nutrient and oxygen competition as well as direct signals by cytokines and metabolites. If correct, this hypothesis would justify therapeutic immune targeting in heart inflammatory disease, a much controversial approach.

A major obstacle to resolve controversy is the lack of clear markers of heart inflammation. Endomyocardial biopsy (EMB), the gold standard for myocarditis diagnosis, includes risks of severe complications (1.5%) and has notoriously poor diagnostic sensitivity (as low as 18% for single, 43% for five biopsies). Cardiac magnetic resonance (CMR) is a powerful tool for detection of acute cardiac inflammation but has limited diagnostic accuracy for chronic inflammatory disease. Critically, CMR cannot identify cause or type of inflammatory infiltrate, features predicting prognosis.

C-reactive protein (CRP) measurement can certainly indicate the presence of an inflammatory process but gives little insight on its location, pathogenesis and stage. Until a breakthrough in the pathophysiology of cardiac inflammation diagnosis is achieved, CRP remains the only, albeit not ideal pathologic cue.

Is it possible that metabolic parameters might reveal more to the clinician than conventional inflammation cues? Reviews of recent trials targeting inflammation in CAD have argued that immunometabolic correction with statins is superior and less prone to severe side effects attached to direct immunomodulation [12]. We have also proposed that modulation of systemic and cellular metabolism might provide an optimal strategy to reduce organ inflammatory disease [7]. To this aim, a systematic analysis of the immunometabolic cross-talk between parenchymal cells and immune cells is paramount to design effective therapeutic approaches.

Here, we summarize the physiological metabolism of heart muscle and provide example of its alteration in paradigmatic heart diseases with a known metabolic/inflammatory pathogenesis. In parallel, we will review key concepts in inflammatory immunometabolism and its potential impact on tissue metabolic steady state and adaptation.

## METABOLIC FLEXIBILITY OF THE HEALTHY HEART

**Figure 1:**
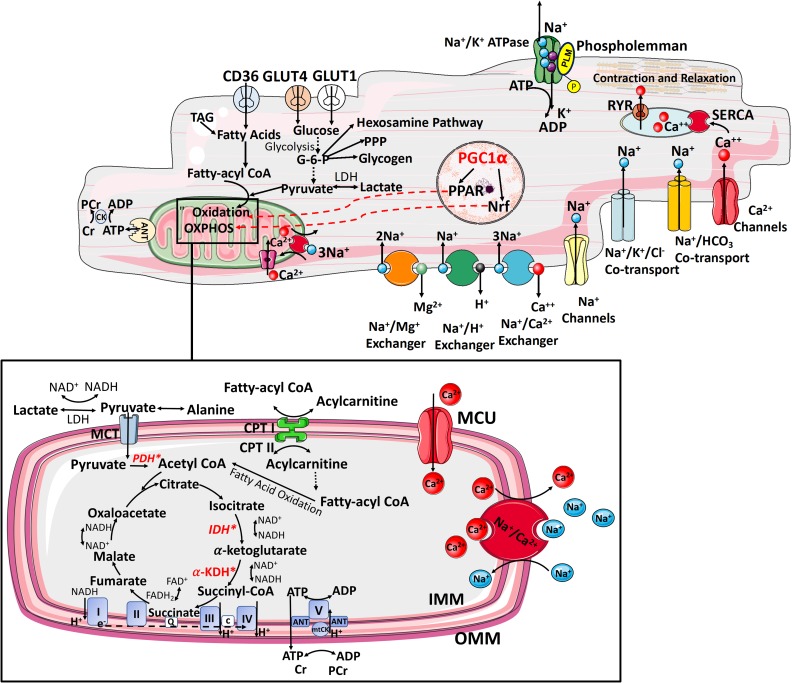
FIGURE 1: Interplay between ATP supply and excitation-contraction coupling in the healthy heart. The delivery of metabolic substrates, their selection and uptake are followed by OXPHOS. It involves electron shuttling from cytosolic to mitochondrial reducing equivalents, transfer of energy by electrons from reducing equivalents to ETC complexes and generation of electrochemical proton (H^+^) gradient within the mitochondrial intermembrane space (respiratory complexes I, II, II, III, IV). The release of H^+^ gradient is coupled to the synthesis of ATP from ADP + P_i_ by F_0_,F_1_-ATPase (complex V), contributing >95% of ATP synthesis under aerobic conditions. The final stage of myocardial ATP supply (phosphotransfer) involves delivery of ATP from mitochondria to sites of use. This involves ADP–ATP exchange across the inner mitochondrial membrane by the adenine nucleotide transporter (ANT) and propagation of local ATP/ADP disequilibria primarily by the creatine kinase (CK) [25]. TAG, triacylglycerol; PCr, phosphocreatine; ANT, adenine nucleotide transporter; GLUT, glucose transporter; CD36, fatty acid transporter; PPP, pentose phosphate pathway; LDH, lactate dehydrogenase; PDH, pyruvate dehydrogenase; CPT, carnitine palmitoyltransferase; CACT, carnitine–acylcarnitine translocase; MCU, mitochondrial calcium uniporter; α-KDH, α-ketoglutarate dehydrogenase; IDH, isocitrate dehydrogenase; mitoCK, mitochondrial creatine kinase; IMM, inner mitochondrial membrane; OMM, outer mitochondrial membrane; Q, quinone pool; c, cytochrome *c*; MPC, mitochondrial pyruvate carrier; e^−^, electrons; *Mitochondrial calcium-sensitive dehydrogenases (pyruvate dehydrogenase, isocitrate dehydrogenase and α-ketoglutarate dehydrogenase) RYR0 ryanodine receptor, SERCA- sarcoendoplasmic reticulum Ca^2+^ ATPase.

The heart is predominantly an aerobic organ and relies on the oxidation of exogenous substrates, such as free fatty acids (FFA), glucose, lactate, ketone bodies, and some amino acids, to generate ATP, the major source of energy. The process of metabolic substrate selection is dynamic and depends largely on substrate availability, O_2_ concentration, and myocardial workload [25, 26]. The heart has an enormous ATP demand— with 2% of its total ATP reserves consumed per beat, it turns over its total ATP pool in less than one min and utilizes six kg of ATP daily [27–29]. Fine control of mitochondrial and cytosolic ATP-generating pathways is critical to meet the energy demands of cardiac muscle [25]. Supply must be matched to demand as failure to provide an adequate amount of ATP causes a decrease in cellular free energy leading to functional decline. Under normal physiological conditions, more than 90% of ATP is generated in mitochondria [29]. Enormous myocardial ATP demand is primarily related to energy-dependent processes driving excitation-contraction (EC) coupling [30] **(Figure 1)**. About 70–75% of total intracellular ATP is used for force generation powering work output, with the remaining 25–30% used for basal metabolism [25].

To synthesize the ATP required to support normal function, the adult heart converts chemical energy primarily stored in FFAs (60–90%) and pyruvate (derived from glucose and lactate 10–40%) into mechanical energy for contraction [31]. There are three principal stages of myocardial ATP supply. The first stage includes metabolic substrate delivery, their selection, uptake and oxidation to generate acetyl-CoA for Kreb's cycle entry. The second stage, OXPHOS, consists of electron shuttling from cytosolic to mitochondrial reducing equivalents (primarily via the malate-aspartate shuttle), transfer of energy by electrons from reducing equivalents to O_2_ [via electron transport chain (ETC) complexes], and generation of an electrochemical proton gradient within the mitochondrial intermembrane space (by complexes I, III, and IV). The release of this gradient is coupled to the synthesis of ATP from ADP by F_0_F1-ATPase (complex V), accounting for 95% of ATP synthesis under aerobic conditions **(Figure 1)**. The third stage is phosphotransfer referring to the delivery of ATP from mitochondria to sites of use **(Figure 1)** [29]. Phosphotransfer involves ADP-ATP exchange across the inner mitochondrial membrane by the adenine nucleotide transporter (ANT) and propagation of local ATP/ADP disequilibria by the creatine kinase shuttle, and to lesser extent by adenylate kinase [32] **(Figure 1)**. However, cardiac workload varies constantly, including several-fold increase in cardiac output during exercise, thus requiring rapid and continuous matching of ATP supply to demand. This renders the heart a metabolic omnivore, giving it a high degree of substrate flexibility to rapidly switch substrate preference and utilization [31]. This is also reflected in the high proportion (>30%) of cardiomyocyte volume occupied by mitochondria and mandated by the low stored ATP content, sufficient to power only ten cardiac cycles, and including phosphotransfer buffer systems, a further 20 cardiac cycles [33].

In order to avoid energetic inefficiency and “waste”, myocardial ATP supply is optimized via the Randle (glucose–fatty acid) cycle which regulates the opposing relationship between carbohydrate and FFA metabolism [34]. FFA enter the cytosol via metabolic transporters, such as FA translocase/CD36 (FAT/CD36), plasma membrane FA binding protein (FABPpm), and FA transport proteins (FATP1 and 6) [35] **(Figure 1)**. In response to stimuli, such as increased insulin or activation of AMP-activated protein kinase (AMPK), FAT/CD36 translocates from intracellular vesicles to the sarcolemma to increase the uptake of FFA [36]. Upon entry into the cytosol, the non-esterified FA are esterified to fatty acyl-CoA. Depending on myocardial demand, fatty acyl-CoA is either stored in the myocardial lipid pool or enters the mitochondria for β-oxidation via the carnitine shuttle carnitine palmitoyl transferase-1 (CPT-1), the rate-limiting enzyme for mitochondrial uptake of FA [35] **(Figure 1)**.

FFA oxidation triggers an increase in mitochondrial ratios of [acetyl-CoA]/[CoA] and [NADH]/[NAD^+^], both of which inhibit the activity of pyruvate dehydrogenase (PDH) complex. Ketone bodies, metabolic products of FFA oxidation in the liver, can also be metabolised to acetyl-CoA for entry into Kreb's cycle [37] **(Figure 1)**.

Myocardial glucose uptake is facilitated by metabolic transporters, principally by the glucose transporter 1 (GLUT1, insulin-independent) and insulin-dependent GLUT4 [38]. Similar to FAT/CD36, in response to stimuli glucose transporters also ‘shuttle' between intracellular vesicles and the sarcolemma. Intracellular glucose is immediately phosphorylated by hexokinase to glucose-6-phosphate, which enters glycolysis, glycogenesis, the pentose phosphate pathway, or the hexosamine biosynthetic pathway **(Figure 1)**. Glycolysis generates a small amount of ATP independent of O_2_ availability, and is regulated mainly by phosphofructokinase, which is inhibited by cytosolic citrate from the Kreb's cycle. Cytosolic citrate is also the major precursor of malonyl-CoA, which inhibits CPT-1 [39]. In normoxia, the end product of glycolysis is pyruvate, which enters mitochondria for oxidation. In hypoxia, pyruvate is reduced to lactate in the cytosol. Mitochondrial PDH is the key enzyme governing the oxidative decarboxylation of pyruvate to acetyl-CoA **(Figure 1)**. Lactate, readily extracted from the bloodstream, can be converted to pyruvate in the cytosol and further metabolized to acetyl-CoA for ATP generation [37]. Arising as the common end product from the oxidation of a variety of substrates, acetyl-CoA enters the Kreb's cycle to produce NADH and FADH_2_, which donate electrons to the ETC thereby creating the proton electrochemical gradient needed to generate ATP. FFA oxidation generates more ATP compared to glucose, but at the expense of greater O_2_ consumption. Therefore, low O_2_ availability drives more metabolically efficient glucose oxidation [31].

By controlling intracellular concentrations of ADP and creatine, the creatine kinase (CK)-mediated phosphotransfer system stimulates both phosphotransfer and OXPHOS flux. This mechanism becomes particularly important at high workloads. Limitations in CK capacity would therefore be expected to limit myocardial contractile reserve at high workloads, assuming O_2_ supply is not limiting. Unlike skeletal muscle, the heart demonstrates stable time-averaged NADH/NAD^+^ as well as concentrations of phosphocreatine and ATP over a wide range of workloads.[40]. A study in the reperfused heart has shown that return of contractile function correlates with ATP turnover, not with [ATP] [41].

### Regulation of cardiac metabolism

Long-term metabolic substrate utilization is governed by the activity of transcription factors that increase or suppress the expression of the key metabolic enzymes. In this context, a pivotal role is played by the peroxisome proliferatoractivated receptors (PPARs), a superfamily of nuclear receptors [42] **(Figure 1)**. PPARα is the member of this family that is most abundantly expressed in the myocardium, where it upregulates the transcription of genes related to FA uptake and oxidation. Similarly, estrogen-related receptorα (ERRα; also known as ESRRA) and ERRγ (also known as ESRRG) target a wide set of genes related to the uptake of metabolic substrates, ATP translocation across mitochondrial membranes, and calcium handling [43] **(Figure 1)**. PPAR activation requires coactivation with PPARγ coactivator 1α (PGC1α) or PGC1β, essential regulators of mitochondrial biogenesis [44], and the binding of PPARs to intermediates of lipid metabolism [45]. Lipid ligands inducing PPARα–PGC1α activation mainly derive from lipolysis of the intracellular triacylglycerol (TAG) pool [46].

### ATP sensing in the heart

AMPK is a stress-activated kinase that functions as a cellular fuel gauge and a critical metabolic regulator. The activity of AMPK is primarily determined by the cellular energy state, reflected in the ratio of AMP (and ADP) to ATP. Activation of AMPK requires the phosphorylation of its α subunit at Thr172 by two upstream kinases, liver Kinase B1 (LKB1) and Ca^2+^/calmodulin-dependent kinase kinase (CaMKK)-β [47]. Once activated, AMPK inhibits various anabolic pathways, including protein synthesis via its action on both mTOR/p70S6K and eEF2 pathways and enhances catabolic pathways, to restore energetic balance required for cell survival [48]. Myocardial AMPK regulates genes related to mitochondrial energy metabolism, including medium chain acyl-CoA dehydrogenase (MCAD), CPT-1, cytochrome C, and uncoupling protein (UCP)-3 [49]. These transcriptional effects are mediated partly by activation of ERRα transcription factor [49]. Furthermore, AMPK regulates peroxisome proliferator activated receptor gamma co-activator (PGC)-1α, a critical modulator of cardiac gene expression and mitochondrial biogenesis. Activated AMPK increases the expression of PGC-1α in hypoxic cardiomyocytes [50]. AMPK phosphorylation of acetyl-CoA carboxylase (ACC2) inhibits its activity and the synthesis of malonyl-CoA, a potent inhibitor of carnitine palmitoyltransferase-1 (CPT-1). The decrease in malonyl-CoA levels, relieves the inhibition of CPT-1, the rate-limiting step for heart fatty acid oxidation [51].

AMPK also stimulates glucose transport and glycolysis. It increases GLUT4 translocation to the sarcolemma by phosphorylating Rab GTPase-activating proteins (GAP) that regulate Rab10, modulators of docking and fusion of GLUT4 vesicles with the plasma membrane [52]. Furthermore, AMPK decreases the endocytosis of GLUT4 leading to increased sarcolemma GLUT4 content and glucose uptake [53]. AMPK acts downstream to glucose transport, by indirectly increasing the activity of phosphofructokinase (PFK)-1, the rate-limiting enzyme in glycolysis. Activated AMPK directly phosphorylates and stimulates PFK-2 to synthesize fructose 2,6-bisphosphate, which in turn allosterically activates PFK-1 [54]. These increases in myocardial glucose transport and glycolysis are important components of the metabolic response to ischemia or hypoxia [55].

## MYOCARDIAL METABOLIC ADAPTION TO STRESS

Myocardial inflammation has emerged as a pathophysiologic process contributing to cardiac hypertrophy, fibrosis and dysfunction in context of heart disease [18, 56, 57]. Accumulating evidence also suggests that myocardial inflammation is also implicated in the development of diabetic cardiomyopathy [58, 59] Several pathological insults can trigger myocardial inflammation which initially represents an adaptive mechanism against stress [18, 56, 57] but becomes maladaptive if stress stimulus persists. Various pathological stressors directly induce the secretion of cytokines, chemokines [interleukin (IL8), monocyte chemoattractant protein-1 (MCP-1)] and adhesion molecules (vascular adhesion molecule 1, intracellular adhesion molecule-1 (ICAM-1)] in cardiomyocytes, fibroblasts and endothelial cells that promote myocardial recruitment of monocytes and lymphocytes [18, 56–58]. However, clear distinction exists between chronic myocardial inflammation and the myocardial inflammation associated with viral myocarditis. Systemic inflammation is subclinical and contributes to the development of cardiac abnormalities in the long term. In contrast, cardiac metabolism can be affected by local inflammatory processes – such as in myocarditis and MI leading to adverse remodeling and HF in rapid manner. Here we summarize two paradigmatic examples of cardiac metabolic adaptation in common conditions of systemic inflammation.

### Metabolism of the failing heart

HF imposes an enormous, worldwide clinical and economic burden, with its increasing prevalence due to progressive ageing of the general population. First identified in the early 20^th^ century, and now a well-established energy starvation hypothesis, it is proposed that maladaptive metabolic remodelling precedes, initiates and maintains adverse contractile dysfunction in hypertrophy and HF [29, 30]. Advances in analytical technologies have improved our insights into the “engine out of fuel” metabolic HF phenomenon and helped to classify metabolic alterations leading to myocardial energy starvation. HF is accompanied by derangements of all three fundamental steps of energy metabolism: substrate uptake and utilization, OXPHOS, and energetics [29]. Using *in vivio* ^31^P nuclear magnetic resonance (NMR), Neubauer and colleagues [29] found that the myocardial phosphocreatine-to-ATP ratio (PCr:ATP) can be used as a reliable prognostic indicator of dilated cardiomyopathy (DCM) where 44% of DCM patients with a PCr:ATP of <1.6 died of cardiovascular causes vs. 5% with a PCr:ATP of >1.6. There have also been numerous preclinical studies as well as clinical data inferring mitochondrial respiratory impairment (complex activities and/or altered expression of the ETC complexes, ATP synthase and adenine nucleotide translocase) in hypertrophy and HF [60–62].

**Figure 2:**
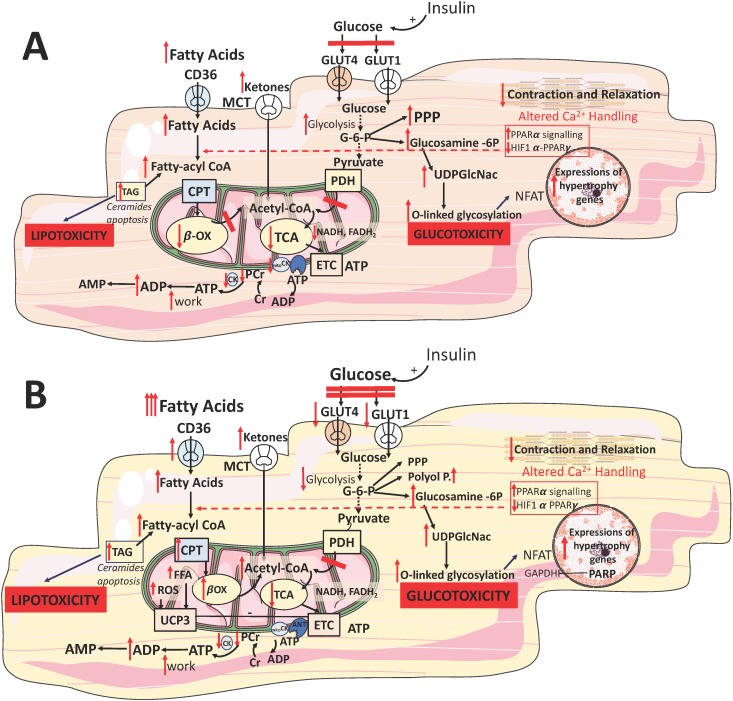
FIGURE 2: Myocardial metabolic adaptation to stress: failing heart (A) and diabetic heart (B). Abbreviations: TAG - triacylglycerol, CPT - carnitine palmitoyl transferase, G6P- glucose 6 phosphate, ETC - electron transport chain, CK - creatine kinase, CD36- fatty acid transporter, Glucosamine 6P - glucosamine 6-phosphate, PPP - pentose phosphate pathway, GLUT - glucose transporter, MCT - monocarboxylate transporter, PDH - pyruvate dehydrogenase, TCA - Kreb's cycle, βOX - beta oxidation, PCr - phosphocreatine, Cr - creatine, NFAT - Nuclear factor of activated T-cells, PPAR - Peroxisome proliferator-activated receptor, ROS - reactive oxygen species, polyol p-polyol pathway. Hypertrophy genes: Class II HDACs, GATA, NFAT, MEF

The size and number of mitochondria are altered in the failing myocardium. There is evidence of mitochondrial misalignment, disorganized cristae, reduced density, membrane disruption and aggregation [63]. Reactive oxygen species (ROS) production from dysfunctional mitochondrial electron transport chain ETC/ATP synthesis intensifies oxidative damage of proteins, lipids and DNA, in a vicious amplifying cycle of mitochondrial dysfunction and ROS production, leading to cardiomyocyte loss [63]. Many studies have explored the relationship between decompensated cardiac remodelling and cardiac substrate utilization. Whereas the question of whether FFA utilization is decreased [64–67] or unchanged [68, 69] during compensated hypertrophy remains subject to debate, the majority of studies reported that the development of overt cardiac dysfunction is accompanied by reduced FFA oxidation [70, 71] **(Figure 2A)**. This metabolic shift was confirmed in multiple animal models of HF, including ischaemic [72] and pacing-induced [73] HF, and was supported by clinical studies reporting a reduced FFA oxidative capacity in HF [74, 75].

Reduced FFA oxidation might be explained at least in part by suppression of PPARα signaling [76, 77] and possibly activation of the hypoxiainducible factor 1α (HIF1α)–PPARγ signalling axis [78], which impairs mitochondrial FFA transport and downregulates the expression of enzymes for FFA oxidation [79] **(Figure 2A)**. However, a metabolic switch away from FFA oxidation is not accompanied by a decrease in myocardial FFA uptake **(Figure 2A)**. Paradoxically, FFA plasma levels are increased in the advanced stages of HF, potentially due to sympathetic activation and consequent increased delivery of FFA to cardiac myocytes [80]. The mismatch between FFA uptake and oxidation leads to intracellular accumulation of lipids [78, 81], which are partly stored as TAGs **(Figure 2A)**, but are also chanelled into non-catabolic pathways, generating toxic lipids, such as ceramide and diacylglycerol (DAG; **Figure 2A)**. This lipotoxicity leads to mitochondrial dysfunction and apoptosis and might contribute to the progression of HF [82, 83]. Furthermore, intracellular accumulation of FFAs contributes to the development of insulin resistance by inducing posttranslational modifications of several components of the insulin signalling cascade [84, 85]. In patients with decompensated HF, mechanical unloading by LV assist device implantation reduced lipid levels and restored myocardial insulin signaling [86]. Thus, collectively there is enough evidence to indicate that oversupply of substrates contributes to the progression of cardiac dysfunction, and insulin resistance has been proposed as the beneficial myocardial mechanism protecting it from the detrimental effects of fuel overload [87].

HF is characterized by a perturbed glucose metabolism: an increase in glucose uptake and glycolytic rates is not accompanied by a concomitant increase in glucose oxidation [71, 88, 89] **(Figure 2A)**. This mismatch between increased glycolytic activity has been observed in the experimental models of cardiac pressure overload [65, 69] and a study in patients with end stage HF [90]. It could be caused by multiple factors: increased activity of the ratelimiting glycolytic enzyme ATPdependent 6phosphofructokinase (PFK1) [91], unchanged or decreased pyruvate oxidation in mitochondria [92], impaired glucose and lactate oxidation related to defective mitochondrial oxidative metabolism [93, 94] and myocardial insulin resistance [93, 95] **(Figure 2A)**. Therefore, although the relative contribution of glucose oxidation to ATP production increases in HF, the absolute substrate flux through glucose oxidative pathways actually decreases. Furthermore, in the late stages of HF cardiac glucose uptake is impaired as a consequence of decreased insulin sensitivity of the myocardium, thereby further reducing the availability of glucose for ATP synthesis [96].

Although a shift towards glucose oxidation in end stage HF could be beneficial given its higher metabolic efficiency than FFA oxidation, it is unlikely to compensate fully for the decrease in FFA utilization. An additional compensatory mechanism in cardiac hypertrophy is anaplerosis — the use of alternative oxidative pathways to generate Krebs cycle intermediates independent of acetyl-CoA [89]. Carboxylation of pyruvate to malate is a major anaplerotic reaction, which might partly account for the mismatch between glycolytic flux and glucose oxidation in mitochondria [89, 97]. However, anaplerotic reactions are energetically less efficient than pyruvate oxidation because they bypass oxidative decarboxylation and other ATP-producing stages of the Kreb's cycle [70].

Two reports have shown an upregulation of enzymes and metabolic intermediates involved in ketone body oxidation in animal models [98] and patients with end stage HF [74], indicating that ketone bodies might become a more relevant energy source in this setting **(Figure 2A)**. Ketone bodies are not readily available from food, but produced in the liver by incomplete oxidation of FFA released from adipose tissue in response to fasting. Because the ATP production:oxygen consumption (P:O) ratio of the ketone body β-hydroxybutyrate (2.50) is higher than that of FFA palmitate (2.33), βhydroxybutyrate was proposed as the ‘super fuel' [99] and that stimulating ketone body over FFA oxidation might be an adaptation to increase myocardial metabolic efficiency. However, this hypothesis is the subject to the same caveat affecting the general myocardial energy starvation model — it assumes that an increase in ATP production efficiency would ameliorate cardiac dysfunction [42]. Consequently, the question of whether an increased utilization of ketone bodies is an adaptive or maladaptive response remains unanswered.

ATP produced by OXPHOS needs to be transmitted to cellular regions of high ATP demand. Inside mitochondria, the phosphate bond of ATP is transferred to creatine by the mitochondrial creatine kinase (mitoCK) to form phosphocreatine (PCr), which rapidly diffuses to the cytosol, where the phosphoryl group is transferred back to ADP by the cytosolic CK **(Figure 2A)** [32]. Thus, the CR-mediated shuttle operates as ATP buffer, preventing an increase in cytosolic ADP by promptly regenerating ATP. These optimized control mechanisms maintain stable levels of high energy phosphates even during haemodynamic challenges to the heart [29]. Many studies using ^31^P-MRS have reported reduced PCr:ATP ratios in patients with HF [100], predicting LV dysfunction and poor outcomes [100]. Strong evidence from the literature indicates that HF is associated with severe impairment of the CK system **(Figure 2A)**, which affects the ATP delivery to the contractile apparatus and ion pumps. However, the results from the mouse models of creatine/CK deficiency challenge the idea that PCr is essential to sustain cardiac function in response to pathological stressors [101]. Thus, the causative role of the CK-mediated phosphotransfer defect in the pathogenesis of HF is still subject to debate.

A number of intracellular signalling pathways have been implicated in the aetiology and regulation of cardiac hypertrophy and HF (reviewed in [102]) including the AMPK-mediated pathway. In chronic pressure overload-induced cardiac hypertrophy, the AMPK activity was shown to increase [103], suggesting that AMPK activation could be necessary compensatory consequence of pressure-overload. This increase in AMPK activity has been suggested to be a major contributor to enhanced glucose uptake in hypertrophy [103], which may be a beneficial metabolic adaptation for an energy-starved heart. However, in contrast, increased AMPK activity in the hypertrophic heart may also contribute to the loss of metabolic flexibility and progression to failure by accelerating FFA oxidation at the expense of glucose oxidation. This would uncouple glycolysis from glucose oxidation, resulting in increased proton production. Increase in proton production is detrimental to the metabolically stressed heart. It leads to accelerated Ca^2+^ overload due to increased Na^+^/H^+^ and Na^+^/Ca^2+^ exchange activities. Subsequently, coupled with alterations in Ca^2+^ handling, larger amounts of ATP are required for the maintenance of intracellular ion gradients thus rendering hypertrophied heart more inefficient by depriving it of valuable ATP needed for the restoration of mechanical function. This may explain why the hypertrophied hearts are more susceptible to ischaemia–reperfusion injury [104, 105]. However, the most recent evidence suggests that AMPK activation counteracts cardiac hypertrophy by reducing O-GlcNAcylation of proteins such as troponin T [48].

### Inflammation and metabolic insufficiency in heart failure - the missing link

In most patients with chronic HF, circulating cytokines are elevated and their levels correlate with the severity of HF and prognosis [106, 107]. Experimental models of HF suggest that pro-inflammatory mediators play an important role in the development and progression of HF [63, 108]. However, many trials of anti-inflammatory therapy for patients with HF have shown neutral or negative effects on outcomes [108].

Impact of cytokines on cardiac function and remodelling in failing heart has been well documented (reviewed in [57, 109]), however, there are very few studies examining the impact on cardiac metabolism. The link between HF and inflammation was first recognized in by Levine *et al.* [110] who reported elevated levels of tumour necrosis factor (TNF) in HF patients with a reduced ejection fraction (EF). Sustained increases in TNF-α have been related to ischaemic myocardial injury, cardiac hypertrophy, and chronic HF. Spontaneously hypertensive rats show increased myocardial TNF-α production, which contributes to remodelling, decreased cardiac function, and faster progression to HF [111]. Likewise, the failing human heart produces large amounts of TNF-α [112], while it has been proposed that persistent intra-cardiac expression of TNF-α contributes to the development of cardiac allograft hypertrophy [113]. Ubiquitous inducible factor named nuclear factor-κB (NF-κB) controls activation of NF-κB itself is involved in various cardiovascular diseases, such as cardiac hypertrophy and HF [113]. Increased TNF-α levels reduce *PGC-1α* and pyruvate dehydrogenase kinase (*PDK4)* expression in human cardiac AC16 cells *in vitro* as well as in heart of TNF1.6 mice, a murine model of cardiac-specific TNF-α overexpression and cytokine-induced cardiomyopathy [112, 114]. A recent *in vitro* study has shown that the p65 subunit of NF-κB directly represses PGC-1α activity in cultured cardiac cells, thereby leading to a reduction in PDK4 expression and the subsequent increase in glucose oxidation observed during the proinflammatory states such as chronic ischaemia, cardiac hypertrophy, and HF [115].

### Metabolic remodeling in diabetic cardiomyopathy

Diabetes is a risk factor for cardiovascular mortality and cardiac remodeling with specific changes to myocardial metabolism, energetics, structure, and function. Diabetic cardiomyopathy is a distinct cardiomyopathy, independent of ischaemia or hypertension, describing the direct effects of systemic diabetes-linked metabolic alterations on myocardial function [116]. Metabolically, diabetes is characterized by rapid defective (type 1 diabetes, T1D) or gradual impairment (type 2 diabetes, T2D) of insulin secretion, leading to increased extracellular glucose and greater reliance on fatty acid oxidation. In both T1D and T2D, failure of insulin to suppress hormone sensitive lipase in adipose tissue and very low-density lipoprotein secretion in the liver increases circulating FFAs [35]. This, in turn, activates PPARα, a transcription factor that upregulates FFA metabolism while decreasing GLUT4, resulting in systemic hyperglycaemia [117]. Early in T2D, the primary problem, the lack of response to insulin in peripheral organs, is over-compensated by increased insulin secretion, resulting in hyperinsulinaemia [118]. Hyperinsulinaemia may be prolonged and may cause a gradual loss of pancreatic function, resulting in hypoinsulinaemia and hyperglycaemia [37]. There is a long-standing idea that insulin resistance and systemic adiposity increase the risk of cardiovascular (CV) events, however a new school of thought is emerging that defines myocardial insulin resistance as a defense against glucotoxicity and oxidative stress [87, 119].

The systemic gradual impairment of insulin production and signalling in diabetes is associated with increased myocardial FFA uptake whilst mitochondrial FFA uptake and oxidation is reduced **(Figure 2B)**. This leads to cytosolic accumulation of TAG, DAG and ceramide **(Figure 2B)** [120]. Such intermediates are pro-apoptotic as they compromise ATP production via the activation of several stress kinases, including protein kinase C (PKC) [121]. PKC inhibits the metabolic action of insulin by phosphorylating the serine/threonine residues on the insulin receptor and/or its substrates [122], disrupting insulin signalling, and inhibiting insulin-stimulated translocation of GLUT4 **(Figure 2B)**. PKC activation triggers apoptosis and leads to lower basal expression of HIF1α and vascular endothelial growth factor [121]. Pharmacological PKC inhibition was shown to ameliorate FFA-mediated inhibition of basal and insulin-stimulated glucose oxidation. It normalized diastolic function in the STZ-treated T1D heart without altering the circulating metabolites [123].

In several clinical studies, proton (^1^H)-MRS has revealed that diabetic patients have between 1.5- and 2.3-fold higher myocardial TAG levels compared to non-diabetic controls, the levels predicting concentric LV remodeling and subclinical, asymptomatic contractile dysfunction [124–126]. Increased availability of plasma FFA increases the flux through myocardial FFA oxidation via activation of the PPARα transcription factor [120, 127]. This leads to the upregulation of enzymes involved in FFA oxidation, including acyl-CoA dehydrogenases **(Figure 2B)**. This metabolic shift is the principal driver of the energetic inefficiency of the diabetic heart. Specifically, unlike glucose oxidation, FFA oxidation requires 11% more O_2_ per carbon. Furthermore, FFA induce expression of mitochondrial uncoupling protein (UCP)-3 through PPAR-α, driving the dissipation of the mitochondrial proton gradient **(Figure 2B)**.

This deteriorates efficiency of ATP production as more O_2_ is required for ATP synthesis, a process defined as mitochondrial uncoupling [128]. A similar concept emerged for UCP2 and UCP3 in HF [98]. In patients undergoing coronary bypass surgery, upregulation of cardiac UCP-3 correlated positively with plasma concentrations of FFA [129] **(Figure 2B)**. The *db/db* mice have increased myocardial UCP-3 that increased mitochondrial inefficiency following ischaemia [130].

In mice, elevation of UCP-3 expression is mediated via increased FFA stimulation of nuclear transcription factor, PPARα [120] **(Figure 2B)**. Activation of the metabolic-sensing ‘master switch' AMPK by metformin was shown to promote both cellular uptake of glucose and β-oxidation of FFAs, attenuating remodeling and HF in diabetes [35]. AMPK also promotes autophagy, providing important nutrients from the breakdown of macromolecules and organelles [131]. In a diabetic mouse HF model, autophagy is impaired and metformin treatment enhances autophagic activity leading to the preservation of cardiac function through an AMPK-dependent mechanism [47]. Nevertheless, animal experiments involving pharmacological activation of PPAR in diabetic hearts remain inconclusive [35], possibly due to the agent specificity for the various PPAR isoforms. Apart for tetradecythioacetic acid (TTA), a PPARα agonist with potent antioxidant properties [132], all other agonists demonstrated reduction in circulating FFA and increased glucose oxidation. Thus, overall cardiac effects were inconsistent: studies employing a PPARγ agonist rosiglitazone and TTA demonstrated improved ischaemic tolerance [131], whereas others using BM17.0744 or 2-(2-(4-phenoxy-2-propylphenoxy)ethyl)indole-5-acetic acid (PPARα and PPARγ agonists, respectively) showed no difference [134, 135].

Acetoacetate and β-hydroxybutyrate (β-OHB) are ketone bodies generated by the liver from non-esterified FAs in response to hypoinsulinaemia and hypoglycaemia. Ketone bodies are oxidized by most body tissues to form acetyl-CoA. Due to its association with life-threatening acidosis in diabetic patients, ketosis has always been a feared status. However, increased ketone metabolism in the diabetic heart has been recently reported [136]. Furthermore, a recent study showed that ketosis may potentially be protective in T2D [128]. In patients with T2D, ketone bodies are more efficient fuel sources than glucose since insulin is not required for their utilization. Therefore, ketogenesis in acute hyperglycaemic crisis may be lifesaving, because it supplies the myocardium with a sufficient amount of ATP [128]. Given that exogenous d-β-hydroxybutyrate, consumed as a ketone ester drink, was metabolized by exercising skeletal muscle to increase endurance performance in athletes and healthy rats [137, 138], it may be that increased ketone metabolism in the diabetic heart is compensating for defects in mitochondrial energy transduction associated with acute insulin deficiency [139].

### Inflammatory signalling promotes metabolic derangement in diabetic cardiomyopathy

Development of myocardial inflammation in diabetes may involve several molecular mechanisms. In principal, these mechanisms converge towards the activation of the NF-κB pathway which is highly active in the diabetic heart and vasculature contributing to damage by promoting the upregulation of cytokines (IL-1β, IL-6, IL-18, TNF-α, TGF-β1), chemokines and adhesion molecules [140–142]. Cardiomyocyte-specific overexpression of IκB-α protein, which suppresses the canonical NF-κB signalling pathway, was observed to prevent streptozotocin-induced diabetic cardiomyopathy through the inhibition of the renin-angiotensin system [143]. Other work also showed that pharmacological inhibition of NF-κB mitigates cardiac oxidative stress induced by T2D and reduces mitochondrial abnormalities [144]. Chronic myocardial inflammation may contribute to cardiac dysfunction by inducing metabolic perturbations that can impair energetics, particularly in response to metabolic stress. Infusion of IL-6 was found to impair cardiac glucose metabolism through a SOCS3-dependent inhibition of IRS-1 [58]. In contrast, genetic disruption of IL-6 gene reduced inflammation and reversed glucose metabolism defects induced by high fat diet, which was paralleled by SOCS3 inhibition and IRS-1 reactivation [145]. Furthermore, cardiac expression of PGC-1α, a master regulator of mitochondrial function and biogenesis [142], was found to be inhibited by a chronic inflammatory process, through a mechanism dependent on NF-κB activation. Exposure of AC16 cells to TNF-α reduced the levels of PGC-1α through the activation of NF-κB [114]. This may be due to p65 binding and sequestering PGC-1α, thereby leading to its inhibition and downregulation [115]. PGC-1α inhibition in response to NF-κB activation was found to cause increased glucose utilization through the downregulation of PDK4 [115].

Hyperglycaemia in T2D also induces cellular damage via four major pathways: activation of the PKC pathway via DAG, increased hexosamine pathway flux, increased advanced glycation end products, and increased polyol pathway flux [146, 147]. All pathways increased ROS production and activated nuclear poly-(ADP-ribose)-polymerase (PARP), which cleaves NAD^+^ into nicotinamide and ADP-ribose [146] **(Figure 2B)**. Overactivation of PARP in hyperglycaemia forces the cell to synthesize NAD^+^ via the salvage pathway which consumes ATP [148]. The process leads to the ribosylation and inactivation of glyceraldehyde-3-phosphate dehydrogenase (GAPDH), which in turn increases glycolytic intermediates and activates the proinflammatory transcription factor NF-κB [146] **(Figure 2B)**. Multiple studies link diabetes to inflammation and it is associated with increased levels of CRP and IL-6 [149]. Other proposed mechanisms for increased inflammation in diabetic cardiomyopathy include oxidative stress via a Ras-related C3 botulinum toxin substrate 1 (RAC1)-mediated activation of NADPH oxidase and endoplasmic reticulum (ER) stress [150].

Many interventions have demonstrated beneficial effects in diabetic cardiomyopathy, attributable in part to reduced cardiac inflammation. These interventions include angiotensin 1 (AT-1) receptor antagonism, activation of the kallikrein–kinin system, inhibition of p38 mitogen-activated protein kinase (MAPK) signalling, gene deletion of kinin receptor b1, inhibition of interleukin converting enzyme, atorvastatin treatment, anti-TNF-α treatment, inactivation of GSK-3β, and cannabidiol treatment ( reviewed in [151]).

## METABOLIC ADAPTION OF IMMUNE CELLS IN INFLAMMATORY MICROENVIRONMENTS

Aerobic glycolysis, i.e. glucose metabolism to lactate in the presence of abundant oxygen, is the preferential pathway adopted by activated and effector immune cells to meet the demands for ATP required for proliferation and the synthesis of inflammatory mediators. As discussed above, although less efficient than oxidative phosphorylation, aerobic glycolysis offers the advantage of a quick release of ATP and a carbon source feeding a range of biosynthetic pathways. Proliferation and differentiation of antigen-activated T lymphocytes is accompanied by metabolic reprogramming towards aerobic glycolysis, anabolic growth and biomass accumulation [152]. Similarly, activated pro-inflammatory myeloid cells mainly utilize glycolysis with minimal oxidative phosphorylation [153–159]. In these cells, interruption of the Kreb's cycle allows the generation of molecules that are important for pro-inflammatory functions [153, 159]. For example, pro-inflammatory macrophages generate high levels of the metabolite succinate, which promotes HIF1α activity and IL-1 production [153]. Increased levels of citrate promote the generation of the antimicrobial metabolite itaconic acid [160, 161]. Dendritic cell (DC) activation leads to a metabolic switch from fatty acid-oxidation and OXPHOS to glycolysis [159], crucial for their antigen-presenting functions [162, 163]. Neutrophils also rely on glycolysis for their effector functions [154, 155, 164], including the formation of neutrophil extracellular traps, which are activated via an mTORC1 signaling pathway [154, 155, 164–166]. Finally, B lymphocytes and natural killer (NK) cells also increase rates of glycolysis in response to various activation stimuli [167–170]. A key molecular mediator of activation-induced glycolysis in most leukocytes is the mammalian target of rapamycin (mTOR) [4, 171]. For example, mTOR complex 1 (mTORC1) activity is essential for induction and maintenance of glycolysis in T cells [4, 172] as well as for cytokine-induced glycolysis in NK cells [167]. Key transcription factors involved in the metabolic reprogramming of immune cells towards glycolysis include HIF1α and c-Myc [172–174]. HIF1α and c-Myc directly induce transcription of genes encoding glycolytic enzymes and glucose transporters.

The development of inflammation leads to dramatic changes in tissue microenvironment, with oxygen and nutrient availability becoming a constraining factor for immune cell function. Albeit this event has not yet been investigated in the context of cardiac inflammation, it is well known that cancerous cells in solid tumours deplete glucose from the microenvironment, thus dampening the ability of adaptive immune cells to utilize glycolysis for their effector functions and promoting the development of an immune-suppressive milieu [8, 175]. Similarly, bacterial infection can lead to local nutrient deprivation due to elevated levels of oxygen and glucose consumption by the invading bacteria [176]. Virus-infected human hepatocytes, fibroblasts and epithelial cells can be reprogrammed to upregulate glycolysis to allow viral replication [177–179]. In parallel to nutrient depletion, inflammation-associated hypoxia arises as the result of multiple factors, including the accumulation of metabolically active leukocytes using aerobic glycolysis, the generation of interstitial oedema leading to an increase in the inter-capillary distance and inflammation-associated fever, which increases oxygen consumption. All of these factors contribute to the development of a hypoxic environment at the inflammatory site, which can in turn modulate the responses of infiltrating immune cells as well as parenchymal cells. Immune cells are able to adapt to oxygen- and nutrient-depleted environment by engaging the HIF1α and AMPK pathways, respectively.

### Immune cell adaptation to hypoxia

HIF1α is a basic helix-loop-helix transcription factor that is strongly induced by hypoxia. In hypoxic conditions, HIF1α binds the constitutively expressed HIF1α (also known as Arnt, the aryl hydrocarbon receptor nuclear transporter), which prevents its degradation and allows its translocation to the nucleus. Here, it binds to hypoxia response elements located in the promoters of a number of genes [180, 181], including glycolytic enzymes and glucose transporters, VEGF, and the chemokine receptor CXCR4.

In addition to hypoxia, HIF1α-mediated transcription can be induced by circumstances associated with pathological stress including cancer, inflammatory mediators, and bacterial infection. HIF1α is expressed by most immune cell populations including macrophages, neutrophils, DCs, as well in T and B lymphocytes and innate lymphoid cells [182]. By regulating immune cell metabolic adaptation to hypoxia, HIF1α plays a key role in modulating their effector functions [183–185]. In physiologically hypoxic environments (i.e. lymph nodes), HIF1α contributes to innate and adaptive immune cell homeostasis, whereas in pathological hypoxia, HIF1α signalling can promote immune cell dysfunction and tissue damage [186].

HIF1α has been implicated in the regulation T-cell development, proliferation, survival, and cytokine production (e.g., IFN-γ), and lack of HIF1α expression has been associated with overproduction of proinflammatory cytokines [187–191]. HIF1α protein stabilization and nuclear translocation can occur in multiple contexts in T cells. While hypoxic exposure of T cells results in modest HIF1α stabilization [192], T cell receptor (TCR) stimulation results in robust HIF1α protein expression, which is further enhanced in hypoxic conditions [192, 193]. TCR-induced HIF1α expression relies upon the PI3 kinase/mTOR pathway [193]. In addition, the pro-inflammatory cytokine IL-6 can induce HIF1α expression in T cells via the STAT3 transcription factor *in vitro* [194]. Of note, HIF1α is not required for the initiation of aerobic glycolysis in activated T cells; instead, c-Myc is uniquely mediating this metabolic shift [172]. However, HIF1α is instrumental in promoting sustained glycolytic responses in Th17 cell differentiation [174]. Th17 cells are induced by IL-6 and TGF-β in a HIF1α-dependent manner [195, 196], while the key transcription factor that drives Th17 differentiation is the retinoic acid-related orphan receptor γt (RORγt) [197]. It has been suggested that the mTOR-HIF1α axis controls Th17 differentiation through transcriptional activation of RORγt and sustained glycolysis [198].

In addition to its role in the development of Th17 responses, HIF1α can also promote the development of anti-inflammatory Tregs thus controlling the homeostatic balance between these T cell subsets with opposite biological effects [174, 190, 191, 194, 199]. Ambient hypoxia (1% oxygen) was shown to induce selective, robust induction of FoxP3, a key transcriptional regulator for Tregs, likely via HIF1α regulation of the FoxP3 promoter. The same study showed that Treg-intrinsic HIF1α is required for optimal Treg function *in vitro* and *in vivo* [200].

Hypoxia and HIF1α stabilization play an important role in B cell development and function. Lack of HIF1α expression in lymphoid tissues of chimeric mice causes abnormal B cell development and autoimmunity [201], due to the impairment of hypoxia-induced cell cycle arrest in B cells [202]. More recently, HIF1α was shown to control the expression of the TASK-2 potassium channels in B cells, which are required for B cell proliferation, survival or cytokine production [203]. HIF1α induces glycolytic metabolism in germinal center (GC) B cells, thus regulating the GC reaction and antibody production [204].

Macrophages are key effectors of innate immunity and – based on their phenotypic and functional features - have been classified into M1 (classic) and M2 (regulatory) macrophages. Classically activated macrophages (M1) play a key role in the innate defense against bacterial infections via the production of large amounts of nitric oxide (NO) by inducible nitric oxide synthase (iNOS) [205]. Alternatively activated (M2) macrophage differentiation is induced by IL-4 and IL-13. These exert anti-inflammatory and pro-angiogenic functions, thus promoting wound healing, tissue repair and regeneration [206, 207]. Macrophage-specific loss of HIF1α expression decreases ATP production, which in turn adversely affects survival, invasion, motility, aggregation and bactericidal activity of murine and human macrophages [208–210]. In addition, mice with myeloid cell-specific deletion of HIF-1α are resistant to lipopolysaccharide (LPS)-induced death [211], suggesting a key role for HIF-1α in classical macrophage polarization [212]. Indeed, LPS-mediated activation and pro-inflammatory (M1) polarization in macrophages perturbs the Krebs cycle, leading to the accumulation of the intermediates fumarate and succinate. These in turn increase HIF1α expression and stabilization, which activates a glycolytic reprogramming promoting the acquisition of a pro-inflammatory phenotype [153, 213–215]. In contrast, alternatively activated (M2) macrophages engage oxidative phosphorylation to meet energy demands [212].

HIF1α stabilization is known to regulate several DC functions, such as survival, differentiation, migration, maturation and antigen presentation. In addition, HIF1α regulates interferon-γ (IFN-γ), IL-22 and IL-10 production in human and murine DCs [216–219]. As in other immune cells HIF1α is involved in metabolic reprogramming of DCs following activation. For example, LPS-induced DC activation under normoxia induces HIF1α expression to greater levels than those induced by hypoxia [220]. HIF1α-induced glycolysis is required for DC maturation [220].

The HIF signaling pathway also increases survival, β2 integrin expression, generation of antimicrobial peptides and glycolytic responses in murine and human neutrophils [208, 209, 221–223].

### Immune cell adaptation to nutrient depletion

Immune cells also have a degree of metabolic plasticity in response to limiting glucose availability. For instance, when glucose levels are low, effector T cells have the ability to adapt and increase glutamine uptake and glutaminolysis to support cellular metabolism [224]. However, in conditions of severe cellular ATP depletion, the nutrient-sensor AMPK becomes activated and induces a catabolic program to preserve survival of immune cells. AMPK is an evolutionarily conserved serine/threonine kinase integrator of energy-sensing signals in the immune system function, which specializes in modulating the cellular responses to an energy challenge [225]. Once activated by stimuli like nutrient deprivation, hypoxia, or excessive ROS production, AMPK activates ATP-producing catabolic pathways while inhibiting ATP-consuming anabolic pathways to restore energy homeostasis. Indeed, AMPK activation is induced by a high cellular AMP / ATP ratio and by upstream kinases [226, 227]. In tumor cells, cardiomyocytes and macrophages/monocytes, AMPK activation boosts the glycolysis rate by increasing glucose uptake and through by phosphorylation and activation of 6-phosphofructo-2-kinase/fructose-2,6-bisphosphatase 2/3, which produces fructose-2,6-bisphosphate, an allosteric activator of glycolytic enzyme phosphofructokinase-1 [228, 229].

In parallel, FA synthesis is reduced via phosphorylation of acetyl-CoA carboxylase (ACC) [230, 231], which catalyzes the rate-limiting step in FA synthesis by converting acetyl-coA to malonyl-coA, and (2) sterol regulatory element-binding protein 1c (SREBP1c), a transcription factor that increases the expression of lipogenic enzymes, including ACC1 and FA synthase [232, 233]. In addition to reducing lipid anabolism, AMPK activates lipid catabolism and mitochondrial biogenesis [229]. Specifically, AMPK increases FA uptake by increasing translocation of the FA transporter CD36 to the plasma membrane [234]. Once inside cells, FAs are transported into the mitochondria for β-oxidation by carnitine palmitoyltransferase-1 (CPT-1). AMPK boosts CPT-1 activity and activates FAO by inducing the inhibitory phosphorylation of ACC2, which is localized to the outer membrane of the mitochondria near CPT-1 where it inhibits production of malonyl-CoA, a potent allosteric inhibitor of CPT-1[233].

AMPK also inhibits glycogen synthesis through inhibitory phosphorylation of glycogen synthase (GS). Furthermore, AMPK also activates glycogen breakdown by phosphorylating and activating glycogen phosphorylase (GP). By indirectly inhibiting mTORC1 through the phosphorylation of TSC2 and raptor, AMPK also inhibits protein synthesis and cap-dependent translation during both initiation and elongation steps [235–237]. Autophagy is a lysosome-dependent self-digestive process activated during nutrient deficiency to preserve cellular integrity. Autophagy is directly initiated by AMPK during severe energy challenges through activation of the Unc-51-like kinase (ULK)-1, a mammalian homolog of ATG1 which removes damaged mitochondria thus maintaining mitochondrial integrity during nutrient starvation [238, 239]. In addition, AMPK indirectly promotes autophagy by inhibiting mTORC1, which phosphorylates ULK1 to prevent interaction with AMPK. Further, AMPK phosphorylates and activates FOXO transcription factors, which in turn upregulate expression of several autophagy inducers, such as Bnip3, LC3 and ATG12 [240].

Although the AMPK and HIF pathways share fundamental functions in the maintenance of cellular metabolic homeostasis, they use different strategies to carry out these functions. Specifically, while HIF factors activate anabolic processes to generate energy, AMPK signalling promotes catabolic mechanisms. In addition, they can control each other's function in a context-dependent manner. For example, HIF1α activates aerobic glycolysis, an effect which is counteracted by AMPK activation [241] possibly via inhibition of protein synthesis [242]. HIF1α and AMPK also exert antagonistic effects in inflammatory microenvironments, e.g. in cancer. HIF-1α signalling has an essential role in the activation and function of immune cells [208, 243]. In contrast, AMPK activation inhibits inflammatory responses by activating the survival factors SIRT1 and FoxO [5, 244]. In addition, the anti-inflammatory cytokines TGFβ and IL10 have been shown to activate AMPK in macrophages via an as yet unidentified mechanism [245].

Substantial evidence supports an indirect, reciprocal regulation between the AMPK and HIF-1α pathways via context-dependent and tissue-selective mediators. For example, AMPK signalling regulates HIF1α nuclear translocation [246], a key step of the hypoxia response. In addition, AMPK is a potent activator of SIRT1, a member of NAD+-dependent sirtuin family, which can either inhibit or activate the signalling of HIF factors. In turn, SIRT1 can directly and indirectly inhibit HIF1α transcriptional activity in cultured cells [247].

Macrophage migration inhibitory factor (MIF) is an evolutionarily conserved, multifunctional cytokine, which has been implicated in the pathogenesis of many cancers and inflammatory diseases [248, 249]. MIF is a secreted pro-inflammatory cytokine expressed not only in immune cells but also in many non-immune cells, such as endothelial and epithelial cells. It is known that this pleiotropic factor transmits signals through a cell surface receptor composed of CD74/CD44 proteins, e.g. activating the ERK1/2, PI3K/Akt, and NF-κB pathways. Interestingly, several studies have demonstrated that the *MIF* gene is a hypoxia-inducible, HIF1α-dependent gene [250–252]. MIF has been shown to activate AMPK signalling eliciting anti-inflammatory effect such as protection from rat heart ischemia reperfusion injury [253], delay of cellular senescence [250, 254] and prevention of liver fibrosis in mice [255]. However, the anti-inflammatory effects of the HIF1α-MIF-AMPK pathway can promote tumorigenesis, known to be induced by MIF [248].

## METABOLIC CROSS TALK BETWEEN IMMUNE CELLS AND CARDIOMYOCYTES DURING INFLAMMATION

Although the immune system has evolved to protect against external stressors including infection, its powerful effector mechanism has the potential to injure and impair function of healthy tissues. Organs with continuous function critical for survival such as eye, brain and the heart are readily impaired by the immune inflammatory responses. Thus, as discussed in section “*CAVEATS AND DEFINITIONS: WHAT IS INFLAMMATORY DISEASE OF THE HEART MUSCLE?*”, the role of inflammation in many cardiac diseases is now widely accepted [58]. There are very few T cells found in healthy cardiac muscle thus maintaining the relative state of immune quiescence which discourages T cell recruitment, activation and presence of resident memory T cells [15]. However, there are significant numbers of resident myocardial macrophages and DCs. The extensive myocardial microvascular network which ensures sufficient oxygen and metabolite supply for continuous cardiac work, provides ample opportunity for circulating T cells to migrate into heart. While direct damage to the myocardium by infiltrating effector immune cells has been widely studied [14], the role of metabolic cross-talk between inflammatory cells and the cardiac parenchyma itself is less well understood.

**Figure 3:**
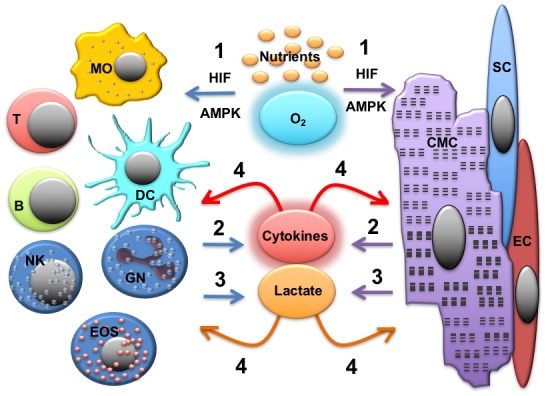
FIGURE 3: Immunometabolic cross-talk in the inflamed heart. During inflammation, inflammatory infiltrates and heart parenchymal cells (including cardiomyocytes, compete with oxygen and nutrients. This leads to the activation of the HIF and AMPK pathways and metabolic reprogramming of both cellular components (1). The production of cytokines (2) and signalling metabolites, such as lactate (3) can lead to further metabolic reprogramming and eventually, to cellular dysfunction (4). MO, macrophages, T, T-lymphocytes; B, B lymphocytes; DC, dendritic cells; NK, NK cells; GN, granulocytes; EOS, eosinophils; CMC, cardiomyocytes; SC, stromal cells; EC, endothelial cells.

As discussed above, both cardiomyocytes and immune cells undergo metabolic adaptation in response to the inflammatory stimuli (cardiac and systemic). As a consequence, these cells can influence each other via the production of soluble mediators and metabolites that can function as signaling molecules **(Figure 3)**.

### Tumor necrosis factor α (TNFα)

Chronic cardiomyocyte exposure to TNFα directly inhibits and downregulates expression of PPAR-γ coactivator-1α (PGC1α) via NFkB (p65) activation [115]. A close relationship exists among PGC-1 function, insulin sensitivity, and T2D with its expression downregulated in T2D subjects [256–258]. A common polymorphism of the PGC1α gene (Gly482Ser), expressing reduced PGC1α activity, increases a risk of T2D [259]. PGC1α is a master regulator of myocardial FFA and glucose oxidative metabolism. It controls expression of transcription factors regulating FFA oxidation, glucose uptake and metabolism, mitochondrial function and biogenesis [259]. PGC1α has been reported to activate the expression of insulin-sensitive GLUT4 [260, 261]. These observations would suggest that reduced levels and compromised activity of PGC1α are driving the metabolic remodelling in cardiac pathologies. The rescue of PGC1α expression by reducing proinflammatory cytokine load could both prevent and ameliorate metabolic remodelling in HF and diabetes: improve mitochondrial function and organisation, reduce insulin resistance by enhancing glucose uptake (GLUT4) and metabolism (PDK4), reducing myocardial steatosis by promoting FFA β-oxidation (due to relationship between myocardial triglyceride accumulation and insulin resistance) [258, 262]. Thus, by altering the nature of immune response we could prevent the origin of pathologic metabolic remodelling (myocardial glucose-FFA oxidation switch), reduced mitochondrial number and activity, as well as rescue metabolic flexibility of hypertrophic heart. However, whether T-cell mediated systemic inflammation significantly contributes to the energetic deficit of HF and diabetes and how the reduction of inflammation by reduction in pro-inflammatory T-cell migration/activation impacts cardiac metabolic (in)flexibility in HF and diabetes needs demonstrating.

### Interleukin-6 (IL-6)

The pro-inflammatory cytokine IL-6 is known for its effect on systemic metabolism [263, 264]. For example, IL-6 promotes FFA mobilization [265], suggesting that IL-6 contributes to shifting metabolic adaptation toward FAO rather than glycolysis. The increase of IL-6 plasma levels during exercise has been proposed to promote energy supply via mobilization of FFA. IL-6 can signal in cardiomyocytes independently of the canonical STAT1/3 pathway, via Erk1/2 and PI3K activation [266]. IL-6R receptors are expressed primarily in myocardial interstitial cells such as fibroblasts and monocytes [267], rather than cardiomyocytes, but are upregulated by cardiomyocytes in response to pro-inflammatory signals as well as mechanical stretch [267, 268]. Overall, IL-6 plays an important protective role in myocardial remodelling in a model of Angiotensin II-dependent hypertension [269] and other IL-6 family cytokines such as leukaemia inhibitory factor (LIF) and cardiotrophin-1 (CT-1) can also promote cardiomyocyte survival and hypertrophy via LIFRβ signalling [270, 271]. However, chronic exposure to IL-6 has been shown to impair myocardial glucose metabolism (via SOC3 inhibition and IRS activation) in diabetic cardiomyopathy in mice [58].

### AMPK-regulating inflammatory cytokines

The traditional paradigm of AMPK as an energy stress-activated kinase has been expanded to include a diverse array of cytokines as AMPK-activators during inflammatory processes [54]. Short-term coronary infusions of pro-inflammatory cytokines such as IL-6, appear to reduce AMPK α subunit content and activation [145]. Fat diet feeding in mice was shown to elevate plasma levels of IL-6, which may contribute to the downregulation of myocardial AMPK seen in this model [145]. MIF is a master-regulator of inflammatory cytokines, and is highly expressed in cardiomyocytes. Hypoxia increases cardiac MIF expression, via a HIF1α-dependent mechanism [272], and ischemia triggers MIF secretion. Endogenous cardiac MIF has an important autocrine-paracrine action to modulate AMPK activation during ischemia and hypoxia in the heart [253]. Extracellular MIF activates AMPK via its cell surface receptor CD74 with subsequent activation of the signal transducer CD44 [253]. MIF knockout mice have impaired heart AMPK activation and are more susceptible to ischemic injury [253].

### Lactate

Highly glycolytic, activated immune cells produce and secrete high amounts of the glycolysis end product lactate in inflamed tissue, including rheumatoid arthritis and cancer [273]. Extracellular lactate can directly signal to immune cells themselves and tissue parenchymal cells via lactate receptors as well as by affecting metabolic pathways when enriched in the cytosol following uptake by lactate transporters.

In immune cells lactate mainly signals via the surface-expressed G-protein-coupled receptor GPR81 [274]. GPR81-mediated signals are strong inhibitors of immune effector functions. For example, extracellular lactate reduces the LPS-induced IL-1β production by murine macrophages and human peripheral blood mononuclear cells (PBMCs) *in vitro* [274]. *In vivo*, high lactate concentrations in the tumour microenvironment contribute to the immune-suppressed microenvironment by inhibiting cytokine production by- and migration of- monocytes and macrophages [275, 276]. Administration of sodium lactate has been shown to reduce the inflammatory response in hepatitis [274] and to prevent the development of inflammation in a colitis model [277]. The anti-inflammatory effects of lactate induced by GPR81 are mediated by inhibition of NF-κB and inflammasome activation [274].

Lactate uptake by immune cells via monocarboxylate transporters (MCTs) can directly affect their cellular metabolism and function independently of GPR8. Expression and modulation of MCTs can control immune cell function in T cells and macrophages [9, 278] by inhibiting the glycolytic pathway [279, 280]. A key enzyme in lactate metabolism is lactate dehydrogenase (LDH), which has a higher affinity for pyruvate compared with lactate. Thus, it converts pyruvate into lactate and NAD^+^. LDH has been implicated in IFNγ-production by T cells [281] and anti-tumour activity of macrophages [282]. Lactate also represents a source of energy for heart muscle by boosting mitochondrial energy metabolism. Lactate amounts to about 10 % of energy production in the healthy heart [283], and this fraction can substantially increase during exercise or different pathophysiological conditions.

In cardiomyocytes lactate production in the cytosol is balanced by oxidation in the mitochondria. Intracellular lactate is shuttled from the cytosol by MCT1 on the mitochondrial membrane [284, 285]. Under normal physiological conditions, low levels of ROS upregulate MCT1 expression by transcriptional mechanisms, thus promoting the transport of lactate into the mitochondria for oxidative metabolism [286]. Not only physical activity but also different pathological conditions could change the hearts' preference from FAs to lactate as energy source [287, 288]. In a rat model of congestive heart failure, e.g., a significant increase in the expression of the lactate transporter MCT 1 was observed [289]. Other studies on rats could demonstrate that an increase in blood lactate level has positive effects on heart function during a hemorrhagic or septic shock [290, 291].

However, excess of extracellular and cytosolic lactate as a result of protracted inflammation has been linked to cardiomyocyte apoptosis in several experimental models of cardiovascular diseases, including myocardial infarction [292], ischemia/reperfusion injury [293], dilated cardiomyopathy [294] and end-stage heart failure [295]. Cardiac MCTs can function as acid loaders or extruders, depending on the transport direction [296], hence the concentration of lactate in the cytosol is directly proportional to the amount of extracellular lactate.

Mechanistically, under pathological conditions, high intracellular lactate concentration promotes the excessive generation of ROS. High levels of ROS can cause oxidative stress and mitochondrial damage, which lead to the activation of mitochondrial-dependent apoptosis [297], a highly regulated program of cell death that can be activated in cardiomyocytes by multiple stressors including cytokines [298], oxidative stress [299] and DNA damage [300]. A significant association has been identified between the lactate signaling cascade and cardiovascular diseases, such as myocardial infarction [284], atrial fibrillation [285] and heart failure [286].

## BREAKING THE VICIOUS CIRCLE: CAN TARGETING METABOLISM REDUCE CARDIAC INFLAMMATION?

As previously discussed, although targeting inflammatory mediators and mechanism in heart disease is a rapidly developing and promising strategy, the lack of diagnostic knowledge of disease-specific mechanisms and stage of inflammation remains a major challenge. On the other hand, several drugs that are currently used in clinic for patients with diabetes, dyslipidemia and metabolic dysfunctions have been shown to impact immune cell function. Understanding the cross-talk between immune cells and myocardium might provide the basis for the repositioning of ‘old' drugs as immunomodulators. Metformin (dimethylbiguanide) widely prescribed for T2D activates AMPK [301], and reduces redox shuttle enzyme mitochondrial glycerophosphate dehydrogenase [302]. Beyond its effects on glucose metabolism, metformin decreases inflammatory markers in plasma, including soluble intercellular adhesion molecule, vascular cell adhesion molecule-1, macrophage migration inhibitory factor, and CRP [303, 304]. The anti-inflammatory effects of metformin are likely related to its ability to inhibit mTOR through AMPK activation [305].

Statins, inhibitors of the enzyme 3-hydroxy-3-methylglutaryl coenzyme A (HMG-CoA) reductase (which catalyzes the formation of mevalonate, the rate-limiting step for cholesterol synthesis), are the most efficient and widely used agents in treating cardiovascular diseases. Originally designed to target elevated lipids, the “traditional” cause of atherosclerosis, statins might also confer cardiovascular benefit by modulating inflammation [306–308]. These effects are independent of the HMG-CoA reductase inhibition [309, 310], while rely upon isoprenoid (and downstream prenylated proteins) biosynthesis from mevalonate [311, 312]. Through this pathway, statins can deviate T-cell differentiation towards the generation of Tregs instead of pro-inflammatory Th17 cells via a mechanism dependent on protein geranylgeranylation [313, 314].

Targeting the PPARs nuclear receptors agonists has also been effective in controlling inflammatory responses in metabolic diseases. In humans, activation of PPARα using fenofibrate or bezafibrate has been shown to decrease plasma levels of several acute phase response proteins that are increased during inflammatory conditions and ameliorates endotoxemia [315]. PPARγ its pharmacological agonists promotes the anti-inflammatory differentiation of macrophages [316, 317] and the function of Tregs in adipose tissue (AT) [318]. Anti-inflammatory effects of PPAR agonists have been reported in a number of model diseases, including inflammatory bowel disease, central nervous system inflammation, LPS-induced cardiac and pulmonary inflammation [319], although the beneficial effect of these drugs in diabetic heart disease remains unclear [35], as previously discussed.

## CONCLUDING REMARKS

This overview of the metabolic plasticity of immune cells and cardiac tissue during inflammation highlights the complexity of immunometabolic events which can determine either the resolution of the inflammatory process or ultimately lead to loss of organ function.

Overall, and perhaps in an over-simplified fashion, the metabolic adaptation of immune cells to the inflammatory microenvironment occurs in synchrony with the evolution of the inflammatory response. Thus, when immune cells localize in the inflammatory site, the hypoxia response mediated by HIF promotes a pro-inflammatory effector function sustained by glycolysis. With the progression of inflammation and increased nutrient consumption relative to supply, the metabolic adaptation mediated by AMPK and lactate favours the development of an anti-inflammatory environment, thus possibly contributing to the resolution of inflammation. The tissue also adapts metabolically and functionally to the changing environment dictated by inflammation in order to cope with damaging events. If this model **(Figure 3)** is correct, it is plausible that abnormal metabolic reprogramming by either immune cells or cardiomyocytes might underlie the progression of inflammation and irreversible tissue damage. As a consequence, pharmacological modulation of abnormal metabolic adaptation may provide an effective approach to treat inflammation-associated heart disease.

## Abbreviations:

AMPK: – AMP-activated protein kinase,
ATP: – adenosine triphosphate,
CAD: – coronary artery disease,
CK: – creatine kinase,
CMR: – cardiac magnetic resonance,
CRP: – C-reactive protein,
DAG: – diacylglycerol,
DC: – dendritic cell,
DCM: – dilated cardiomyopathy,
ERR: – estrogen-related receptor,
ETC: – electron transport chain,
FA: – fatty acid,
FFA: – free FA,
GC: – germinal center,
HFpEF: – HF with preserved ejection fraction,
HIF: – hypoxia-inducible factor,
IL: – interleukin,
LDH: – lactate dehydrogenase,
LV: – left ventricular,
LPS: – lipopolysaccharide,
MCT: – monocarboxylate transporter,
MI: – myocardial infarction,
MIF: – migration inhibitory factor,
mTOR: – mammalian target of rapamycin,
NK: – natural killer,
OXPHOS: – oxidative phosphorylation,
PARP: – poly-(ADP-ribose)-polymerase,
PCr: – phosphocreatine,
PDH: – pyruvate dehydrogenase,
PFK: – phosphofructokinase,
PKC: – protein kinase C,
PPAR: – peroxisome proliferator-activated receptor,
ROS: – reactive oxygen species,
T1D: – type 1 diabetes,
T2D: – type 2 diabetes,
TAG: – triacylglycerol,
TCR: – T cell receptor,
TNF: – tumor necrosis factor,
Treg: – regulatory T cell,
TTA: – tetradecythioacetic acid.
